# The Identification of Novel Prognostic and Predictive Biomarkers in Breast Cancer via the Elucidation of Tumor Ecotypes Using Ecotyper

**DOI:** 10.1002/cai2.70013

**Published:** 2025-05-27

**Authors:** Feng Du, Jie Ju, Fangchao Zheng, Songlin Gao, Peng Yuan

**Affiliations:** ^1^ Key Laboratory of Carcinogenesis and Translational Research (Ministry of Education/Beijing), The VIPII Gastrointestinal Cancer Division of Medical Department Peking University Cancer Hospital and Institute Beijing China; ^2^ Key Laboratory of Carcinogenesis and Translational Research (Ministry of Education/Beijing), Department of Day Care Peking University Cancer Hospital and Institute Beijing China; ^3^ Department of Medical Oncology, Cancer Research Center, Shandong Cancer Hospital and Institute Shandong First Medical University and Shandong Academy of Medical Sciences Jinan Shandong Province China; ^4^ Department of VIP Medical Services, National Cancer Centre/National Clinical Research Center for Cancer/Cancer Hospital Chinese Academy of Medical Sciences and Peking Union Medical College Beijing China

**Keywords:** breast cancer, intra‐tumor heterogeneity, prognosis

## Abstract

**Background:**

Breast cancer is a highly heterogeneous disease, characterized by tumor and nontumor cells at various cell states. Ecotyper is an innovative machine learning framework that quantifies the tumor microenvironment and delineates the tumor ecosystem, demonstrating clinical significance. However, further validation is needed in breast cancer.

**Methods:**

Ecotyper was applied to identify multiple cellular states and tumor ecotypes using large‐scale breast cancer bulk sequencing data, followed by a detailed analysis of their associations with clinical classification, molecular subtypes, survival prognosis, and immunotherapy response. Identified subtypes were further characterized using single‐cell and spatial data sets to reveal molecular profiles.

**Results:**

In a comprehensive analysis of 6578 breast cancer samples from four data sets, Ecotyper identified 69 cellular states and 10 tumor ecotypes. Of these, 37 cellular states significantly correlated with overall survival. Notably, specific states within epithelial cells, macrophages/monocytes, and fibroblasts were linked to a worse prognosis. CE2 abundance was identified as the most significant marker indicating unfavorable prognosis and was further validated in an additional data set of 116 HER2‐negative patients. These biomarkers also indicated the efficacy of neoadjuvant immunotherapy in breast cancer. CE2‐high cancers were characterized by an abundance of basal‐like epithelial cells, scant lymphocytic infiltration, and activation of hypoxia signaling. Single‐cell analysis showed that CE2‐high areas were rich in SPP1‐positive tumor‐associated macrophages(TAM), basal‐like epithelial cells, and hypoxic cancer‐associated fibroblasts(CAF). Spatially, these regions were often peripheral in triple‐negative breast cancer, adjacent to fibrotic/necrotic zones. Multiplex immunofluorescence confirmed the enrichment of SPP1+CD68+TAM and HIF1A+SMA+CAF in hypoxic triple‐negative breast cancer (TNBC) regions.

**Conclusions:**

Ecotyper identified novel biomarkers for breast cancer prognosis and treatment prediction. The CE2‐high region may represent a hypoxic immune‐suppressive niche.

## Graphical abstract

A novel breast cancer subtyping based on intra‐tumoral microenvironment heterogeneity predicts prognosis and immunotherapy response. The CE2 subtype, in particular, is linked to the worst outcomes and the most significant immunotherapy benefits, with distinct cellular and molecular features mediated by hypoxia.

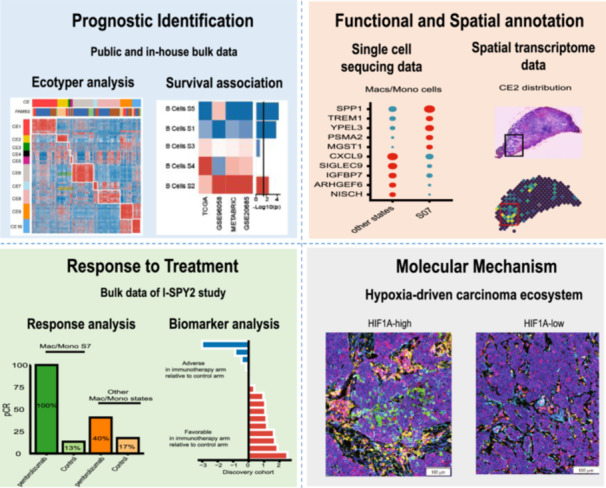

**Abbreviations**
CAF: cancer‐associated fibroblast
CE: carcinoma ecotype
CS: cell state
ITH: intra‐tumor heterogeneity
Mono/Macs: monocyte/macrophage
scRNA‐seq: single‐cell RNA sequencing
ST: spatial transcriptome
TAM: tumor‐associated macrophage
TME: tumor microenviroment
TNBC: triple‐negative breast cancer

## 1 Introduction

Breast cancer is a highly heterogeneous disease, exhibiting significant diversity in the genetic background, spatial distribution, and interactions between tumor and nontumor cells [1, 2, 3, 4]. For decades, the heterogeneity of breast cancer was primarily characterized by the features of malignant epithelial cells, using methods such as the PAM50 classification [1, 5, 6]. However, this strategy overlooks nonmalignant cellular components and thus may oversimplify the complexity of tumors. The tumor microenvironment (TME) is composed of diverse cell types, such as stromal and immune cells, extracellular matrix components, and signaling molecules, and it fosters tumor growth and resistance through intricate interactions among these components [7]. Emerging evidence has revealed TME as a pivotal driver of cancer progression, influencing metastasis and therapeutic response [8, 9]. In addition to tumor cells, immune and stromal cells also play crucial roles in tumor progression [10, 11, 12]. Recent studies in breast cancer demonstrated that the characteristics of the TME significantly impact the prognosis of breast cancer patients and the tumor response to immunotherapy [13, 14, 15, 16, 17]. The interactions between malignant and nonmalignant cells promote tumor development and may serve as potential targets for intervention. Therefore, deciphering the complex interplay between cancer cells and the TME is essential for understanding cancer progression and addressing therapeutic challenges. The study of distinct cell functions within the immune microenvironment, such as cytotoxic T lymphocytes [7] and gamma delta T cells [18], has gained momentum. The heterogeneity, regulatory mechanism, and functional roles of these cell types and strategies for their enhancement in antitumor immunity are now at the forefront of current research.

Previous classifications have primarily focused on inter‐tumoral heterogeneity but have not adequately captured the features and clinical significance of intra‐tumoral heterogeneity (ITH). In recent years, with the rapid advancement of technologies such as single‐cell transcriptomics and proteomics, novel classifications have been proposed. The discovery of the carcinoma ecosystem, which refers to the complex interactions between cancer cells and their surrounding microenvironment, has further revealed the characteristics of ITH and its potential mechanisms. Recently, a novel single‐cell intrinsic subtyping system was developed that can identify recurrent patients in breast cancer [19]. Additionally, a specific ecosystem associated with immune suppression and poor prognosis was also identified using mass cytometry [20]. Because of technological and cost limitations, validating these subtypes in large‐scale samples is challenging, making it difficult to assess their clinical significance.

Recently, the emergence of Ecotyper, a new machine learning framework, has made it possible to validate the significance of the carcinoma ecosystem in large numbers of clinical samples [21]. Ecotyper is a versatile and cross‐platform analytical framework that enables multi‐dimensional analyses by three steps: computationally refining cell‐type‐specific gene expression profiles, identifying and quantifying transcriptionally defined cell states, and assigning these states to multicellular communities. Ecotyper allows for the discovery and recovery of consistent cellular states (CS) and carcinoma ecotypes (CE) across bulk, single‐cell and spatial transcriptomics data, which facilitates comprehensive analyses across different dimensions and demonstrates significant potential for clinical utility [21, 22].

In this study, we applied this framework in the study of breast cancer, with the goals of identifying novel prognostic biomarkers and biomarkers that predict immunotherapy response and exploring the landscape of ITH in breast cancer.

## 2 Materials and Methods

### 2.1 Study Population and Patient Cohorts

#### 2.1.1 CAMS Cohort

A total of 116 patients from the Cancer Hospital, Chinese Academy of Medical Sciences (CAMS) who were diagnosed with Stage I–III breast cancer from 2009 to 2017 were included. All patients underwent breast mastectomy and received adjuvant chemotherapy after surgery.

#### 2.1.2 Data Sets

The following data sets were analyzed in this study: The Cancer Genome Atlas (TCGA)‐BRCA cohort of 998 patients with RNA‐seq data and complete follow‐up information; The Molecular Taxonomy of Breast Cancer International Consortium (METABRIC cohort), including 1980 patients with microarray data; the GSE96058 cohort, with 3273 patients with RNA‐seq data; the GSE20685 cohort, with 327 patients with microarray data and raw CEL data collected and processed as previously described [23]; the Wolf cohort (GSE194040, 997 patients with RNA‐seq data); the Pusztai cohort (GSE173839, 105 patients with RNA‐seq data); the Wu cohort (scRNA‐seq data and cell annotation file: GSE176078, including 100,064 cells from 26 breast cancer patients); and ST data, with pre‐processed 10X Visium ST data and tumor annotation files of two breast cancer cases with triple‐negative breast cancer (CID44971 and 1142243F).

### 2.2 Data Processing

#### 2.2.1 In‐House Data RNA Sequencing

RNA sequencing was performed on formalin‐fixed paraffin‐embedded tumor samples from the 116 patients in the CAMS cohort. RNA purification, reverse transcription, library construction, and sequencing were performed at Sequanta Technologies Co. Ltd in Shanghai, China. Sequencing was conducted on an Illumina Novaseq. 6000 platform (Illumina, San Diego, USA), following Illumina‐provided protocols for 2 × 150 paired‐end sequencing.

#### 2.2.2 Single‐Cell and ST Data Analysis

Seurat objects were constructed using Seurat (version 5.0.0). We applied quality control filters to remove cells that expressed fewer than 200 genes and genes that were detected in fewer than 3 cells. We normalized data using “NormalizeData” and “ScaleData” function. All cells in the specific Ecotype assigned cell state were compared with the rest of the cell types in the data by the “FindMarkers” function to display the differentially expressed genes for each particular cell state with the threshold of 0.05 of p_val_adj. Spatial feature expression plots were generated with the “SpatialFeaturePlot” function to visualize the data.

#### 2.2.3 Ecotyper Analysis

Briefly, the Ecotyper approach incorporates gene expression profiles derived from 12 specific cell types as a reference and builds a learning model to apply it to external data sets. When analyzing bulk transcriptome data, the “Recovery of Cell States and Ecotypes in User‐Provided Bulk Data” module was applied, which allowed us to obtain the relative abundances (as continuous variables) of 69 cell transcriptional states for 12 main cell types. Ecotyper assigned the most significant cell state (as a categorical variable) to each sample. By analyzing the co‐occurrence patterns of different cell states, Ecotyper generated 10 distinct tumor ecological types [7]. In the case of single‐cell sequence data, the “Recovery of Carcinoma Cell States and Ecotypes in scRNA‐seq Data” module was used to assign cell states (as categorical variables) to each cell. For spatial transcriptome data, the “Recovery of Cell States and Ecotypes in Spatial Transcriptomics Data” module was used to obtain the abundance of each cell state and ecotype in each spot. The scripts and vignette are provided at https://github.com/digitalcytometry/ecotyper [7].

### 2.3 Characterizing the Function of Cellular States and Carcinoma Ecotypes

A total of 44 gene sets covering cell composition, immune features, and tumor pathway‐related genes were obtained from previous studies [16, 20, 24]. For annotation of carcinoma ecotypes, we performed enrichment analysis on each sample using the “gsva” method from the GSVA package (v1.46.0) [25]. We conducted AUCcell (1.20.2) [26] on single‐cell sequencing data to generate the enrichment scores of angiogenesis and hypoxia signature.

### 2.4 Survival Analysis

We used univariate and multivariate Cox regression analyses using the coxph function from the survival package (v3.5.5) to assess the survival correlations of each CS and CE abundance. The weighted z‐value was calculated using the formula as described [27], taking into account the inverse square root of the sample size of each study as weights. All survival weighted z‐values were converted to two‐sided −log10 *p* values for clarity. Survival associations are expressed as −log10 *p*‐values oriented by survival direction. Kaplan–Meier analysis, implemented through the survfit function in the survival package, was used to estimate overall survival and for discrete variables such as carcinoma ecotype assignments. The two‐sided log‐rank test method was used for significance calculation.

### 2.5 Multiplex Immunofluorescence Staining

The PANO 7‐plex IHC kit (catalog number 0004100100; Panovue, Beijing, China) was used for multiplex immunofluorescence staining. Fluorescent imaging of slides was conducted with an Olympus VS200 scanner equipped with a 20X UPLXAPO objective lens (both from Olympus Germany). The images were analyzed using QuPath software.

### 2.6 Data Analysis and Statistics

Statistical analysis for differences in continuous variables among multiple groups was performed using the Kruskal–Wallis rank sum test. Differences in categorical variables among multiple groups were assessed using the chi‐square test. All data and figures were analyzed using R software (version 4.2.2). *p* < 0.05 indicated statistical significance.

## 3 Results

### 3.1 Prognostic Atlas of Cellular States Across Breast Cancer

**Figure 1:**
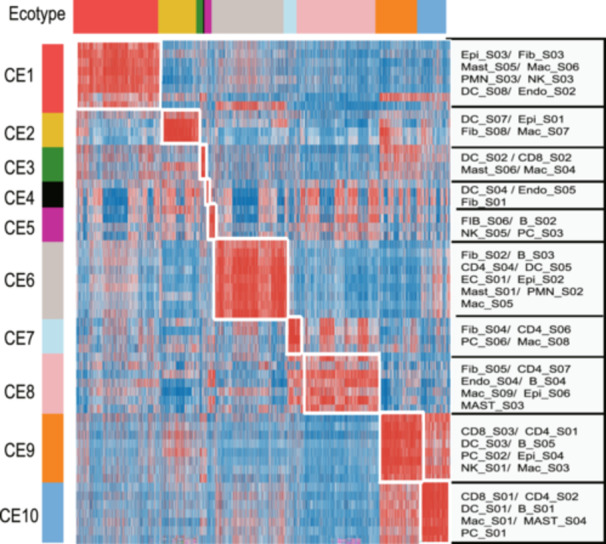
Cellular state abundance profiles across four data sets, organized into 10 carcinoma ecotypes (CEs). Only cellular states and tumor samples assigned to CEs are shown. Tumor samples are ordered by the most abundant CE class per specimen.

We gathered bulk sequencing data from four independent data sets, which included 6,578 breast cancer cases with comprehensive clinical follow‐up details (Table S1). A total of 69 cellular states were recovered in each patient by Ecotyper, covering most cellular types within the TME, including epithelial cells, CD8 T cells, CD4 T cells, natural killer cells, B cells, plasma cells, mast cells, monocytes/macrophages, dendritic cells, fibroblasts, endothelial cells, and neutrophils. Each type of cell was represented by 3–9 cellular states, for which we obtained both abundance (continuous variables) and assignment (categorical variables) (Figure 1).

**Figure 2:**
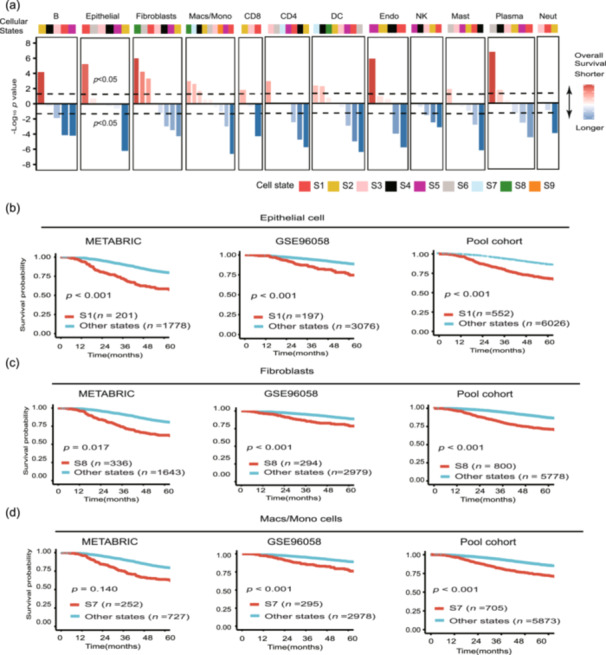
Cell‐state‐specific survival associations across 6578 tumors. (a) Cellular state survival associations of 69 cellular states in 6578 breast tumors, stratified by cell type and aggregated across data sets. Colors indicate favorable (blue) or adverse (red) survival. (b–d) Kaplan–Meier plots showing differences in overall survival between patients assigned to (b) epithelial cell state 1 (S1) or other epithelial cellular states, (c) fibroblasts state 8 (S8) or other fibroblast states and (d) macrophage/mono state 7 (S7) or other macrophage/mono states. Patients were stratified by assigning each tumor to its most prevalent state per cell type. The pooled cohort included all patients in TCGA, METABRIC, GSE96058, and GSE20685 data sets. Statistical significance was calculated by a two‐sided log‐rank test.

We used Cox regression analysis to correlate the cellular state abundance with overall survival. In univariate analysis, most cellular states (42/69) demonstrated markedly prognostic association. After adjusting for ER status, HER2 status, TNM staging, or lymph node status, 54% (37/69) maintained prognostic significance (Figure 2a, Figure S1, Tables S2 and S3). The direction of survival association was generally consistent across the four data sets for most cellular states.

After assigning cell states as categorical variables for survival analysis, we found that each of the epithelial cells, fibroblasts, and macrophages/monocytes exhibited a cellular state that was significantly associated with a worse prognosis (Figure 2a–d). These findings were validated in two independent data sets (METABRIC and GSE96058) and the pooled cohorts, highlighting the potential of Ecotyper in identifying novel prognostic markers in breast cancer.

### 3.2 Characterization of CEs in Breast Cancer

**Figure 3:**
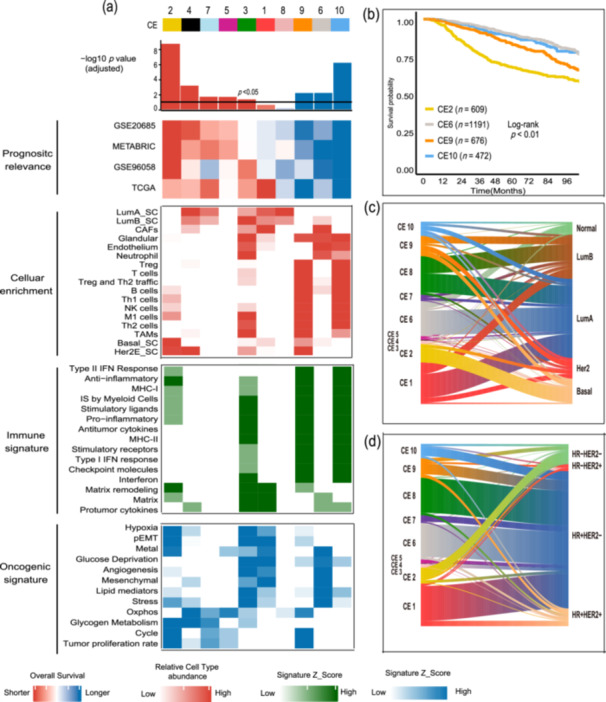
The survival association and characteristics of carcinoma ecotypes (CE). (a) Characteristics of 10 CEs. Top: Multi‐variable associations with overall survival (OS); Center: Enrichment of cell type specific signature classified into CE‐specific subgroups on the basis of the most prevalent CE per sample; Bottom: Enrichment of immune and oncogenic signatures in each CE subgroup. (b) Kaplan–Meier plot for OS for patients assigned to the selected prognostic CEs. (c) Overlap between 10 CEs and 5 PAM50 subtypes on all evaluable bulk BRCA tumors with labels from both classification schemes available. (d) Overlap between 10 CEs and 4 IHC‐based subtypes on all evaluable bulk BRCA tumors with labels from both classification schemes available.

Ecotyper formulates 10 CEs on the basis of the interplay of multiple cellular states. We derived abundance and assignment for each ecotype in every patient. Univariate and multivariable survival analyses of CE abundance revealed that 8 out of 10 CEs showed significant prognostic relevance (Figure 3a, Figure S2a). CE2 exhibited the strongest negative correlation with prognosis and is most pronounced in the HER2‐positive and basal‐like subtypes (Figure S2c). Kaplan–Meier analysis also showed significant survival differences when treating CE as categorical variables (Figure 3b, Figure S2b).

To illustrate CE2 composition and functionality, we selected 44 signatures encompassing cellular components and TME features [16, 20, 24]. The CE2 subtype showed upregulation of the basal‐like epithelial signature (Figure 3a), aligning with its clinical phenotype. Most CE2 cases were classified as either basal‐like subtype or triple‐negative breast cancer (TNBC) (Figure 3c–d). The CE2 subtype also exhibited a lack of lymphocyte signaling and instead showed elevated levels of immune suppression and stromal remodeling signals (Figure 3a). CE2 tumors exhibited high levels of tumor proliferation, hypoxia, partial epithelial‐mesenchymal transition, and altered glucose metabolism (Figure 3a).

**Figure 4:**
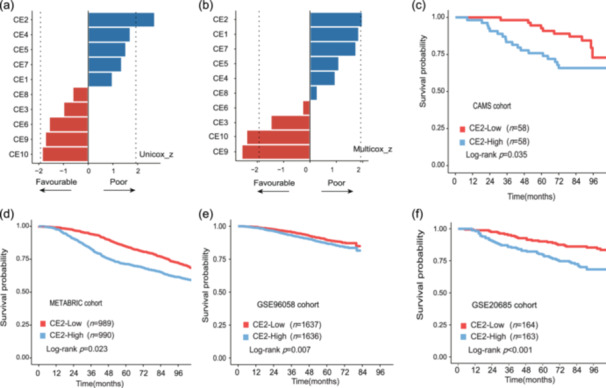
The prognostic significance of CE2 abundance. (a–b) The (a) univariate and (b) multivariate survival analysis of 10 carcinoma ecotype (CE) abundance in the Cancer Hospital of Chinese Academy of Medical Sciences (CAMS) cohort. Color indicates favorable (red) or adverse (blue) survival. The dotted line represents that the z‐value has statistical significance in Cox regression analysis. (c–f) The Kaplan–Meier plot of patients with high and low abundance of CE2 in (c) the CAMS cohort, (d) METABRIC cohort, (e) GSE96058 cohort, and (f) GSE20685 cohort. Patients were divided by the median of CE2 abundance. Statistical significance was calculated by a two‐sided log‐rank test.

To further validate the prognostic value of CE2 abundance, we performed Ecotyper analysis on the 116 HER2‐negative breast cancer patients from the CAMS cohort. Both univariate and multivariable survival analyses demonstrated the association of CE2 with worse prognosis (Figure 4a–b). Stratifying patients by CE2 abundance in in‐house or public data sets revealed consistent results, showing that CE2‐high patients had unfavorable prognosis (Figure 4c–f). These findings indicated the reliability of CE2 abundance as a novel prognostic marker for breast cancer.

### 3.3 The Association of Cellular States and Community With Immunotherapy Therapeutic Benefit

**Figure 5:**
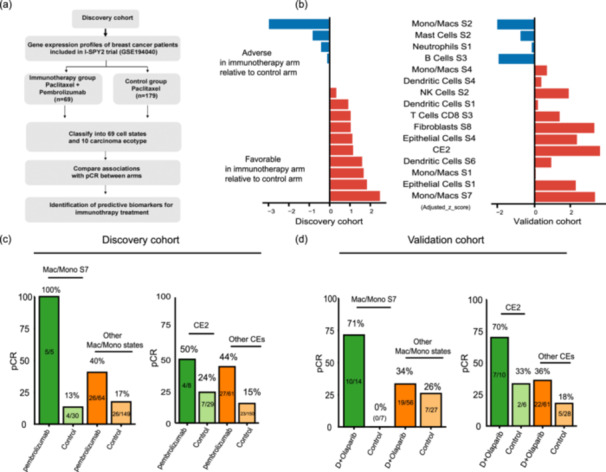
Identification of predictive markers of immunotherapy by Ecotyper. (a) Outline of the approach to identify predictive markers. (b) Association between selected markers and therapeutic benefit from chemotherapy and pembrolizumab relative to chemotherapy in the discovery and validation cohorts. Cellular states were ranked by an adjusted z‐score of univariate logistic regression analysis that penalizes associations with pathological complete response in chemotherapy. (c–d) The pathological complete response rate bar plots for patients stratified by treatment arm and macrophage/monocellular states or carcinoma ecotype subtype in (c) the discovery cohort and (d) validation cohort.

The TME profoundly influences immunotherapy outcomes. Ecotyper quantitatively dissects the complex milieu of the TME, offering prospects for discovering predictive biomarkers. The ISPY‐2 trial is an adaptive platform study designed to identify and validate biomarkers that predict response to neoadjuvant chemotherapy in patients with breast cancer. We examined 2 of the 10 treatment arms in this study: the control group comprising 179 patients who received sequential paclitaxel followed by doxorubicin plus cyclophosphamide, and the experimental group consisting of 69 patients who underwent the same chemotherapy regimen in combination with pembrolizumab [28]. All patients were HER2‐negative, and the outcome was pathological complete response (pCR) (Figure 5a).

We used EcoTyper for the quantification of 69 cellular states and 10 carcinoma ecotypes in all patients, followed by univariate logistic regression analysis to evaluate the correlation with pCR within each arm. Using the established algorithm [22], we calculated the adjusted z‐score of each biomarker, which assigned higher values to states that predict a greater therapeutic benefit from the treatment of pembrolizumab plus chemotherapy than solely chemotherapy.

Further validation was done in the GSE173839 data set, which consists of 34 patients who received chemotherapy and 71 HER2‐negative patients who received chemotherapy plus durvalumab and olaparib [29]. Figure 5b displays the markers that demonstrated a consistent trend with the net immunotherapy benefit across both data sets.

Notably, the EPIS1, FIBS8, Macs/Mono S7, and CE2 abundance demonstrated the same trend towards greater net immunotherapy benefit. When treating them as categorical variables, patients assigned to Macs/Mono S7 exhibited higher pCR rates with immunotherapy compared with those in the control group or in other Macs/Mono states. The same pattern was observed in patients assigned to the CE2 subtype (Figure 5c–d). In the GSE173839 data set, CE2 demonstrated superior predictive ability for pCR following immunotherapy compared with conventional biomarkers such as tumor mutational burden and PD‐1 expression (Figure S3 **).** These results highlighted the potential of Ecotyper in identifying predictive biomarkers for immunotherapy.

### 3.4 Hypoxia May Drive the Emergence of CE2‐high Tumors

CE2 was the parent ecotype of EPIS1, FIBS8, and Macs/Mono S7, which indicated that these unfavorable cellular states constituted a detrimental ecosystem with significant clinical implications (Figure 1). Therefore, we further explored the potential key factors behind this phenomenon.

**Figure 6:**
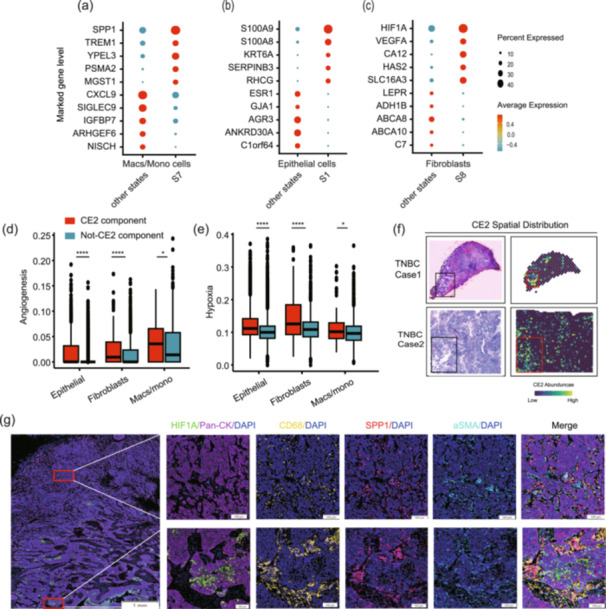
Hypoxia may regulate the formation of CE2. The specific marker genes of CE2‐specific (a) macrophage/monocyte state 7, (b) epithelial state 1, and (c) fibroblast state 8. The (d) angiogenesis and (e) hypoxia signature score of CE2‐specific component and others. *p < 0.05, ****p < 0.0001. Kruskal–Wallis rank sum test. (f) The spatial distribution of CE2 in two triple‐negative breast cancer cases from Wu et al. [20]: (Case 1: CID44971, Case 2: 1142243F). (g) Immunofluorescence imaging of CE2‐specific markers (DAPI, PAN‐CK, CD68, SPP1, αSMA, and HIF1A) in triple‐negative breast cancer specimen from the CAMS cohort.

First, we used the well‐annotated data set of GSE176078 and assigned each cell to the types defined by Ecotyper. Differential expression marker analysis revealed that Macs/Mono S7 cells specifically expressed SPP1 and TREM1, indicating similarity to SPP1+ tumor‐associated macrophages (TAMs) (Figure 6a). Fibroblast S8 cells exhibited high expression of HIF1A and VEGFA, suggesting similarity to hypoxia‐associated cancer‐associated fibroblasts (CAFs) (Figure 6b). Epithelial S1 cells displayed specific expression patterns related to cell keratinization and basal epithelial cells (Figure 6c). We then conducted functional analysis on the cellular states and observed significant activation in angiogenesis and hypoxia‐related pathways in these cells compared with other cells, indicating the involvement of hypoxia in forming CE2‐high tumors (Figure 6d–e).

We next used Ecotyper with spatial transcriptomics data from two TNBC cases. The CE2‐high region was closer to the tumor edge and tended to be surrounded by stroma or necrotic tissue (Figure 6f **)**. We then used multiplex immunofluorescence to detect the expression of HIF1A, SPP1, PAN‐CK, CD68, and ɑSMA in six TNBC patients. The results indicated that regions with high HIF1A expression were correlated with an increased accumulation of SPP1+CD68+TAMs and HIF1A+ɑSMA+CAFs, suggesting the role of hypoxia in promoting the formation of the aggressive multicellular community and thus accelerating cancer progression in TNBC (Figures 6g and S4).

## 4 Discussion

In this study, we identified four biomarkers that exhibited prognostic relevance in large sample sizes from both public and in‐house data sets using Ecotype. Furthermore, we discovered a strong link between the biomarkers and the response to immunotherapy. This study underscored the capabilities of Ecotyper in dissecting the breast cancer microenvironment and identifying reliable clinical markers.

Previous studies have demonstrated that specific cellular components in a particular state within the TME can predict patient prognosis and treatment outcomes [16, 30]. Ecotyper offers a convenient method for quantitative analysis of the TME, enabling researchers to extract rich data and gain deep insights from bulk sequencing results. In pan‐cancer and lymphoma research, Ecotyper has shown robust capabilities in screening prognostic markers [21, 22]. Ecotyper has also identified new tumor subtypes in studies on sarcoma and colorectal cancer [31, 32]. In this study, we used Ecotyper and identified three clinically relevant cellular components in breast cancer. Notably, the cell population CE2, composed of these three cellular components, also exhibits significant clinical and biological characteristics. This finding suggests that tumor cells, macrophages, and fibroblasts in breast cancer may interact with each other to jointly promote tumor progression.

Recent studies have indicated that cells within the TME are not randomly distributed but form distinct cellular communities under specific driving forces. Bill et al. [33] discovered that tumor macrophage polarity identifies a highly coordinated network of pro‐ or antitumor variables. This network involves each tumor‐associated cell type and is spatially organized. Another study revealed the concomitant response of several adjacent cell types to the same TME features, such as cells of several cell types colocalizing in highly proliferative niches [34]. In this study, we identified three cellular states with poor prognosis that collectively formed the CE2 TME niche, which influenced tumor progression and immunotherapy response. CE2 may thus be a critical component of the breast cancer microenvironment.

Our findings indicate that hypoxia may be a pivotal factor in CE2 formation. Hypoxic signaling is elevated in the cellular elements of the CE2 niche. Additionally, an enrichment of SPP1+ TAMs and hypoxic fibroblasts was noted in the tumor's hypoxic areas. Previous studies reported that hypoxia induces upregulation of SPP1 expression in both tumor cells and macrophages in breast cancer [35], and SPP1+ TAMs engage with nearby cells via cellular communication, fostering tumor growth [36, 37, 38].

This study has several limitations. First, we used the typing method embedded in Ecotyper and conducted a detailed analysis of molecular typing and therapeutic efficacy tailored to breast cancer, which produced outcomes distinct from those in prior pan‐cancer studies. Second, the reliability of the predictive biomarkers needs to be confirmed in prospective cohorts. Third, the specific interaction mechanisms among the cellular components in the CE2 subtype warrants further experimental investigation.

## 5 Conclusion

This study applied Ecotyper to discover new prognostic and predictive biomarkers, characterize the breast cancer ecosystem, and offer insights for more precise risk stratification and immunotherapy patient selection. These results enhance the understanding of ITH and its clinical relevance.

## Author Contributions

**Feng Du:** methodology (lead), software (lead), visualization (lead), writing – original draft (lead). **Jie Ju:** data curation (supporting), resources (supporting). **Fangchao Zheng:** methodology (equal), writing – review & editing (equal). **Songlin Gao:** methodology (supporting), software (supporting). **Peng Yuan:** conceptualization (lead), resources (lead), supervision (lead).

## Ethics Statement

This study was approved by the Independent Ethics Committee of National Cancer Centre/Cancer Hospital, Chinese Academy of Medical Sciences and Peking Union Medical College Hospital (approval number: 23/139‐3884).

## Consent

Written informed consent was obtained from all participants.

## Conflicts of Interest

The authors declare no conflicts of interest.

## Supporting information

supplementary figures 1‐4‐final version.

Supplementary table.xlsx.

## Data Availability

RNA‐seq and clinical information of TCGA‐BRCA cohort were downloaded from GDC TCGA Cancer in UCSC Xena. The METABRIC data were downloaded from http://www.cbioportal.org/. The expression data and clinical information of GSE96058, GSE194040, GSE173839, and GSE20685 were collected from Gene Expression Omnibus (GEO). The scRNA‐seq data and cell annotation were collected from GEO (https://www.ncbi.nlm.nih.gov/geo/query/acc.cgi?acc=GSE176078). The ST data and tumor annotation file were downloaded from https://www.zenodo.org/record/4739739. The RNA‐seq data of the CAMS cohort are available from the corresponding author upon reasonable request.

## References

[1] N. Zhao and J. M. Rosen , “Breast Cancer Heterogeneity Through the Lens of Single‐Cell Analysis and Spatial Pathologies,” Seminars in Cancer Biology 82 (2022): 3–10, 10.1016/j.semcancer.2021.07.010. 34274486 PMC8761219

[2] L. Guo , D. Kong , J. Liu , et al., “Breast Cancer Heterogeneity and Its Implication in Personalized Precision Therapy,” Experimental Hematology & Oncology 12, no. 1 (2023): 3, 10.1186/s40164-022-00363-1. 36624542 PMC9830930

[3] J. Zhai , Y. Wu , F. Ma , V. Kaklamani , and B. Xu , “Advances in Medical Treatment of Breast Cancer in 2022,” Cancer Innovation 2, no. 1 (2023): 1–17, 10.1002/cai2.46. 38090370 PMC10686187

[4] F. Zheng , F. Du , J. Zhao , et al., “The Emerging Role of RNA N6‐Methyladenosine Methylation in Breast Cancer,” Biomarker Research 9, no. 1 (2021): 39, 10.1186/s40364-021-00295-8. 34044876 PMC8161983

[5] M. Cregger , A. J. Berger , and D. L. Rimm , “Immunohistochemistry and Quantitative Analysis of Protein Expression,” Archives of Pathology & Laboratory Medicine 130, no. 7 (2006): 1026–1030, 10.5858/2006-130-1026-iaqaop. 16831029

[6] J. S. Parker , M. Mullins , M. C. U. Cheang , et al., “Supervised Risk Predictor of Breast Cancer Based on Intrinsic Subtypes,” Journal of Clinical Oncology 27, no. 8 (2009): 1160–1167, 10.1200/jco.2008.18.1370. 19204204 PMC2667820

[7] S. Peng , A. Lin , A. Jiang , et al., “CTLS Heterogeneity and Plasticity: Implications for Cancer Immunotherapy,” Molecular Cancer 23, no. 1 (2024): 58, 10.1186/s12943-024-01972-6. 38515134 PMC10956324

[8] L. Liu , Y. Xie , H. Yang , et al., “HPVTIMER: A Shiny Web Application for Tumor Immune Estimation in Human Papillomavirus‐Associated Cancers,” iMeta 2, no. 3 (2023): e130, 10.1002/imt2.130. 38867938 PMC10989930

[9] A. Glaviano , H. S. Lau , L. M. Carter , et al., “Harnessing the Tumor Microenvironment: Targeted Cancer Therapies Through Modulation of Epithelial‐Mesenchymal Transition,” Journal of Hematology & Oncology 18, no. 1 (2025): 6, 10.1186/s13045-024-01634-6. 39806516 PMC11733683

[10] V. Thorsson , D. L. Gibbs , S. D. Brown , et al., “The Immune Landscape of Cancer,” Immunity 48, no. 4 (2018): 812–830.e14, 10.1016/j.immuni.2018.03.023. 29628290 PMC5982584

[11] W. Chen , L. Shen , J. Jiang , et al., “Antiangiogenic Therapy Reverses the Immunosuppressive Breast Cancer Microenvironment,” Biomarker Research 9, no. 1 (2021): 59, 10.1186/s40364-021-00312-w. 34294146 PMC8296533

[12] F. Du , F. Zheng , Y. Han , J. Zhao , and P. Yuan , “Novel Immune‐Related Gene Signature for Risk Stratification and Prognosis of Survival in ER (+) and/or PR (+) and HER2 (–) Breast Cancer,” Frontiers in Pharmacology 13 (2022): 820437, 10.3389/fphar.2022.820437. 35721151 PMC9201983

[13] J. A. Joyce , “Therapeutic Targeting of the Tumor Microenvironment,” Cancer Cell 7, no. 6 (2005): 513–520, 10.1016/j.ccr.2005.05.024. 15950901

[14] H. Ahmed , A. R. Mahmud , M. F. R. Siddiquee , et al., “Role of T Cells in Cancer Immunotherapy: Opportunities and Challenges,” Cancer Pathogenesis and Therapy 1, no. 2 (2023): 116–126, 10.1016/j.cpt.2022.12.002. 38328405 PMC10846312

[15] R. C. M. C. Silva , M. F. Lopes , and L. H. Travassos , “Distinct T Helper Cell‐Mediated Antitumor Immunity: T Helper 2 Cells in Focus,” Cancer Pathogenesis and Therapy 1, no. 1 (2023): 76–86, 10.1016/j.cpt.2022.11.001. 38328613 PMC10846313

[16] A. Bagaev , N. Kotlov , K. Nomie , et al., “Conserved Pan‐Cancer Microenvironment Subtypes Predict Response to Immunotherapy,” Cancer Cell 39, no. 6 (2021): 845–865.e7, 10.1016/j.ccell.2021.04.014. 34019806

[17] Y. Bareche , L. Buisseret , T. Gruosso , et al., “Unraveling Triple‐Negative Breast Cancer Tumor Microenvironment Heterogeneity: Towards an Optimized Treatment Approach,” JNCI: Journal of the National Cancer Institute 112, no. 7 (2020): 708–719, 10.1093/jnci/djz208. 31665482 PMC7357326

[18] Z. Gao , Y. Bai , A. Lin , et al., “Gamma Delta T‐Cell‐Based Immune Checkpoint Therapy: Attractive Candidate for Antitumor Treatment,” Molecular Cancer 22, no. 1 (2023): 31, 10.1186/s12943-023-01722-0. 36793048 PMC9930367

[19] E. Azizi , A. J. Carr , G. Plitas , et al., “Single‐Cell Map of Diverse Immune Phenotypes in the Breast Tumor Microenvironment,” Cell 174, no. 5 (2018): 1293–1308.e36, 10.1016/j.cell.2018.05.060. 29961579 PMC6348010

[20] S. Z. Wu , G. Al‐Eryani , D. L. Roden , et al., “A Single‐Cell and Spatially Resolved Atlas of Human Breast Cancers,” Nature Genetics 53, no. 9 (2021): 1334–1347, 10.1038/s41588-021-00911-1. 34493872 PMC9044823

[21] B. A. Luca , C. B. Steen , M. Matusiak , et al., “Atlas of Clinically Distinct Cell States and Ecosystems Across Human Solid Tumors,” Cell 184, no. 21 (2021): 5482–5496.e28, 10.1016/j.cell.2021.09.014. 34597583 PMC8526411

[22] C. B. Steen , B. A. Luca , M. S. Esfahani , et al., “The Landscape of Tumor Cell States and Ecosystems in Diffuse Large B Cell Lymphoma,” Cancer Cell 39, no. 10 (2021): 1422–1437.e10, 10.1016/j.ccell.2021.08.011. 34597589 PMC9205168

[23] A. J. Gentles , A. M. Newman , C. L. Liu , et al., “The Prognostic Landscape of Genes and Infiltrating Immune Cells Across Human Cancers,” Nature Medicine 21, no. 8 (2015): 938–945, 10.1038/nm.3909. PMC4852857 26193342

[24] D. Barkley , R. Moncada , M. Pour , et al., “Cancer Cell States Recur Across Tumor Types and Form Specific Interactions With the Tumor Microenvironment,” Nature Genetics 54, no. 8 (2022): 1192–1201, 10.1038/s41588-022-01141-9. 35931863 PMC9886402

[25] S. Hänzelmann , R. Castelo , and J. Guinney , “GSVA Gene Set Variation Analysis for Microarray and RNA‐Seq Data,” BMC Bioinformatics 14, no. 1 (2013): 7, 10.1186/1471-2105-14-7. 23323831 PMC3618321

[26] S. Aibar , C. B. González‐Blas , T. Moerman , et al., “SCENIC: Single‐Cell Regulatory Network Inference and Clustering,” Nature Methods 14, no. 11 (2017): 1083–1086, 10.1038/nmeth.4463. 28991892 PMC5937676

[27] M. C. Whitlock , “Combining Probability From Independent Tests: The Weighted Z‐Method Is Superior to Fisher's Approach,” Journal of Evolutionary Biology 18, no. 5 (2005): 1368–1373, 10.1111/j.1420-9101.2005.00917.x. 16135132

[28] D. M. Wolf , C. Yau , J. Wulfkuhle , et al., “Redefining Breast Cancer Subtypes to Guide Treatment Prioritization and Maximize Response: Predictive Biomarkers Across 10 Cancer Therapies,” Cancer Cell 40, no. 6 (2022): 609–623.e6, 10.1016/j.ccell.2022.05.005. 35623341 PMC9426306

[29] L. Pusztai , C. Yau , D. M. Wolf , et al., “Durvalumab With Olaparib and Paclitaxel for High‐Risk HER2‐Negative Stage II/III Breast Cancer: Results From the Adaptively Randomized I‐SPY2 Trial,” Cancer Cell 39, no. 7 (2021): 989–998.e5, 10.1016/j.ccell.2021.05.009. 34143979 PMC11064785

[30] J. Wagner , M. A. Rapsomaniki , S. Chevrier , et al., “A Single‐Cell Atlas of the Tumor and Immune Ecosystem of Human Breast Cancer,” Cell 177, no. 5 (2019): 1330–1345.e18, 10.1016/j.cell.2019.03.005. 30982598 PMC6526772

[31] A. Subramanian , N. Nemat‐Gorgani , T. J. Ellis‐Caleo , et al., “Sarcoma Microenvironment Cell States and Ecosystems Are Associated With Prognosis and Predict Response to Immunotherapy,” Nature Cancer 5, no. 4 (2024): 642–658, 10.1038/s43018-024-00743-y. 38429415 PMC11058033

[32] S. Li , T. Pan , G. Xu , et al., “Deep Immunophenotyping Reveals Clinically Distinct Cellular States and Ecosystems in Large‐Scale Colorectal Cancer,” Communications Biology 6, no. 1 (2023): 785, 10.1038/s42003-023-05117-1. 37500893 PMC10374645

[33] R. Bill , P. Wirapati , M. Messemaker , et al., “CXCL9: SPP1 Macrophage Polarity Identifies a Network of Cellular Programs That Control Human Cancers,” Science 381, no. 6657 (2023): 515–524, 10.1126/science.ade2292. 37535729 PMC10755760

[34] A. Gavish , M. Tyler , A. C. Greenwald , et al., “Hallmarks of Transcriptional Intratumour Heterogeneity Across a Thousand Tumours,” Nature 618, no. 7965 (2023): 598–606, 10.1038/s41586-023-06130-4. 37258682

[35] S. Chen , C. Liao , H. Hu , et al., “Hypoxia‐Driven Tumor Stromal Remodeling and Immunosuppressive Microenvironment in Scirrhous HCC,” Hepatology 79, no. 4 (2024): 780–797, 10.1097/hep.0000000000000599. 37725755

[36] J. Qi , H. Sun , Y. Zhang , et al., “Single‐Cell and Spatial Analysis Reveal Interaction of FAP+ Fibroblasts and SPP1+ Macrophages in Colorectal Cancer,” Nature Communications 13, no. 1 (2022): 1742, 10.1038/s41467-022-29366-6. PMC8976074 35365629

[37] Y. Liu , Z. Xun , K. Ma , et al., “Identification of a Tumour Immune Barrier in the HCC Microenvironment That Determines the Efficacy of Immunotherapy,” Journal of Hepatology 78, no. 4 (2023): 770–782, 10.1016/j.jhep.2023.01.011. 36708811

[38] G. Fan , T. Xie , L. Li , L. Tang , X. Han , and Y. Shi , “Single‐Cell and Spatial Analyses Revealed the Co‐Location of Cancer Stem Cells and SPP1+ Macrophage in Hypoxic Region That Determines the Poor Prognosis in Hepatocellular Carcinoma,” NPJ Precision Oncology 8, no. 1 (2024): 75, 10.1038/s41698-024-00564-3. 38521868 PMC10960828

