# Supplementary material for: The Identification of Novel Prognostic and Predictive Biomarkers in Breast Cancer via the Elucidation of Tumor Ecotypes Using Ecotyper
Source: Cancer Innov. 2025 May 27;4(4):e70013. doi: 10.1002/cai2.70013 (PMC12107130; doi:10.1002/cai2.70013)
Supplement: Supplementary file 1 — supplementary figures 1‐4‐final version. [file CAI2-4-e70013-s001.pdf]

Supplementary Figure legend

Fig S1. Univariate cell state survival associations in four independent cohorts. colored by favorable(blue) or adverse (red) survival

Fig S2. (a) Univariable associations of carcinoma ecotypes with OS **(b)** The Kaplan-Meier plot of 10 carcinoma ecotypes in the pooled cohort.**(c)** Multivariate carcinoma ecotypes survival associations in each PAM50 subtype **(d)** Bar chart showing the carcinoma ecotypes distribution among normal and tumor patients in the TCGA-BRCA dataset.

Fig S3. The ROC curves of CE2 and other biomarkers for predicting pCR following immunothera py in the GSE173839 dataset.

Fig S4. Box plot showing the differences of **(a)** CD68+SPP1+ and **(b)** HIF1A+ αSMA+ cell ratio in HIF1A-high versus low regions of TNBC tumors from six patients.

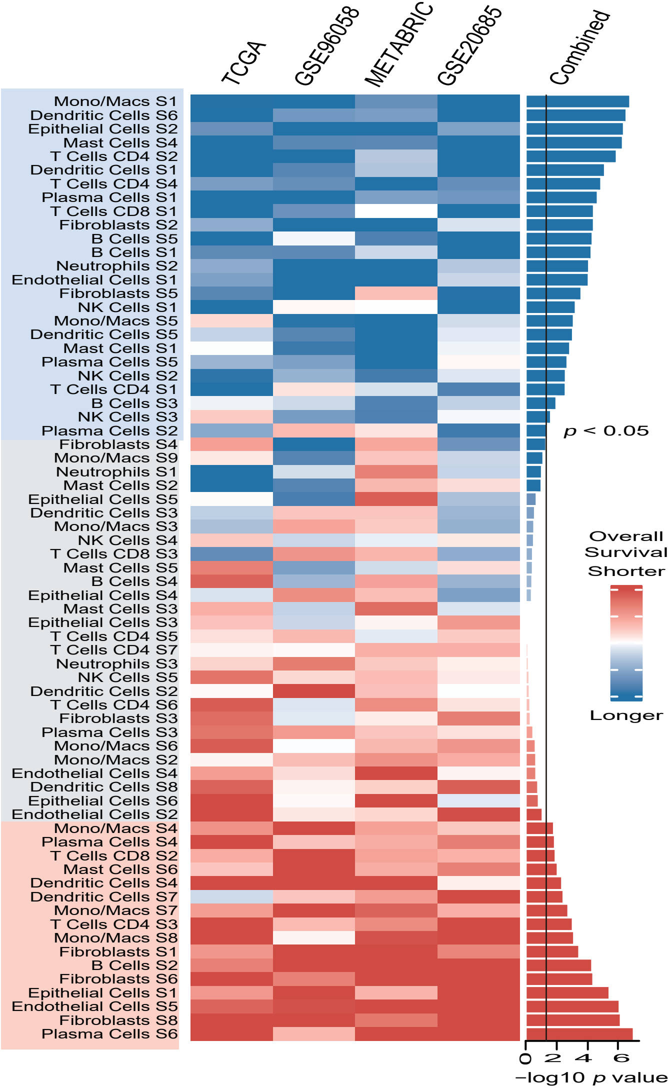
TCGA
GSE96058
METABRIC
GSE20685
Combined
Mono/Macs S1
Dendritic Cells S6
Epithelial Cells S2
Mast Cells S4
T Cells CD4 S2
Dendritic Cells S1
T Cells CD4 S4
Plasma Cells S1
T Cells CD8 S1
Fibroblasts S2
B Cells S5
B Cells S1
Neutrophils S2
Endothelial Cells S1
Fibroblasts S5
NK Cells S1
Mono/Macs S5
Dendritic Cells S5
Mast Cells S1
Plasma Cells S5
NK Cells S2
T Cells CD4 S1
B Cells S3
NK Cells S3
Plasma Cells S2
Fibroblasts S4
Mono/Macs S9
Neutrophils S1
Mast Cells S2
Epithelial Cells S5
Dendritic Cells S3
Mono/Macs S3
NK Cells S4
T Cells CD8 S3
Mast Cells S5
B Cells S4
Epithelial Cells S4
Mast Cells S3
Epithelial Cells S3
T Cells CD4 S5
T Cells CD4 S7
Neutrophils S3
NK Cells S5
Dendritic Cells S2
T Cells CD4 S6
Fibroblasts S3
Plasma Cells S3
Mono/Macs S6
Mono/Macs S2
Endothelial Cells S4
Dendritic Cells S8
Epithelial Cells S6
Endothelial Cells S2
Mono/Macs S4
Plasma Cells S4
T Cells CD8 S2
Mast Cells S6
Dendritic Cells S4
Dendritic Cells S7
Mono/Macs S7
T Cells CD4 S3
Mono/Macs S8
Fibroblasts S1
B Cells S2
Fibroblasts S6
Epithelial Cells S1
Endothelial Cells S5
Fibroblasts S8
Plasma Cells S6
p < 0.05
Overall
Survival
Shorter
Longer
0
2
4
6
−log10 p value

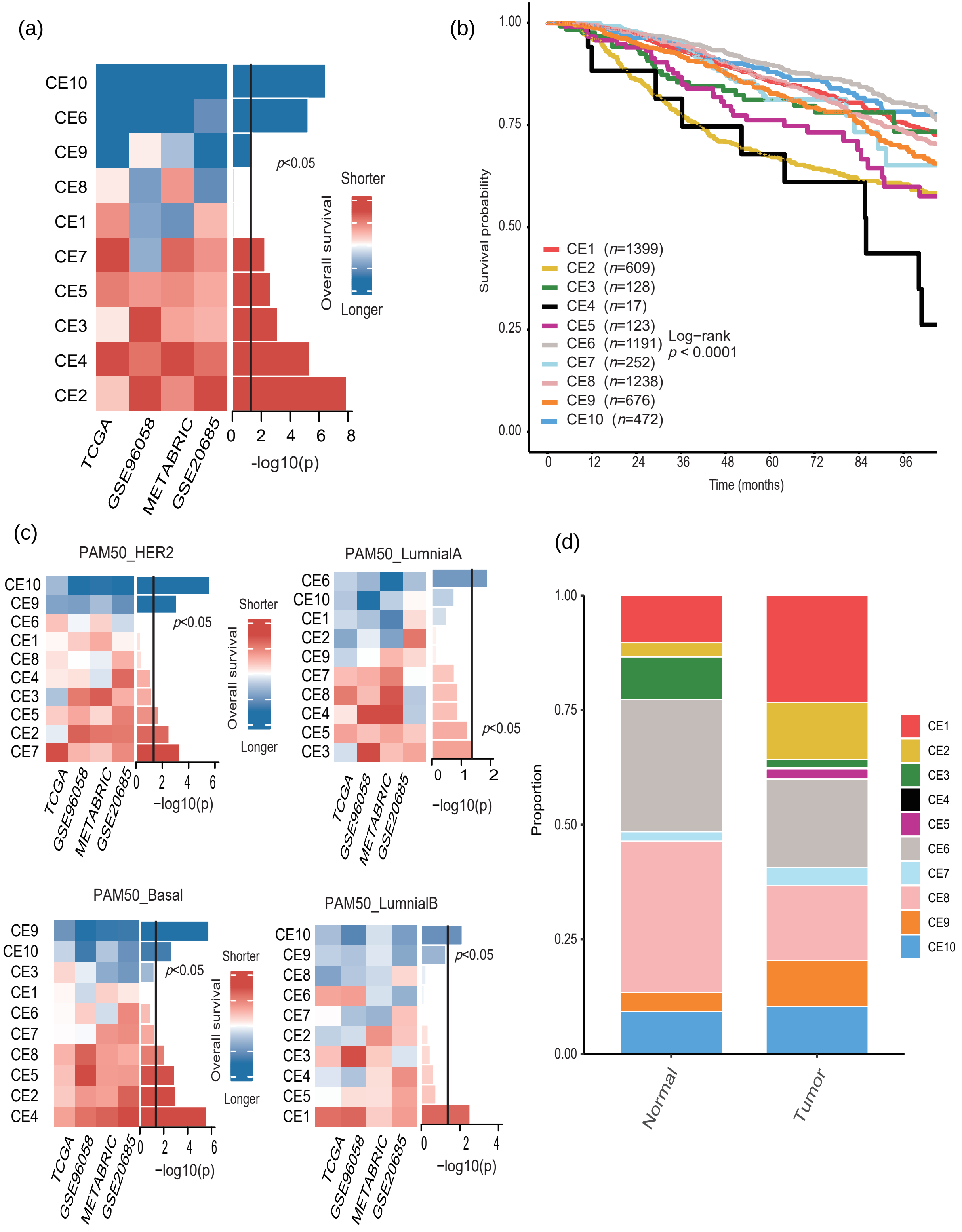

(a)
CE10
CE6
CE9
CE8
CE1
CE7
CE5
CE3
CE4
CE2
TCGA
GSE96058
METABRIC
GSE20685
p<0.05
-log10(p)
Shorter
Overall survival
Longer
(b)
Survival probability
Time (months)
CE1 (n=1399)
CE2 (n=609)
CE3 (n=128)
CE4 (n=17)
CE5 (n=123)
CE6 (n=1191)
CE7 (n=252)
CE8 (n=1238)
CE9 (n=676)
CE10 (n=472)
Log−rank
p < 0.0001
(c)
PAM50_HER2
PAM50_LumnialA
PAM50_Basal
PAM50_LumnialB
(d)
Proportion
Normal
Tumor

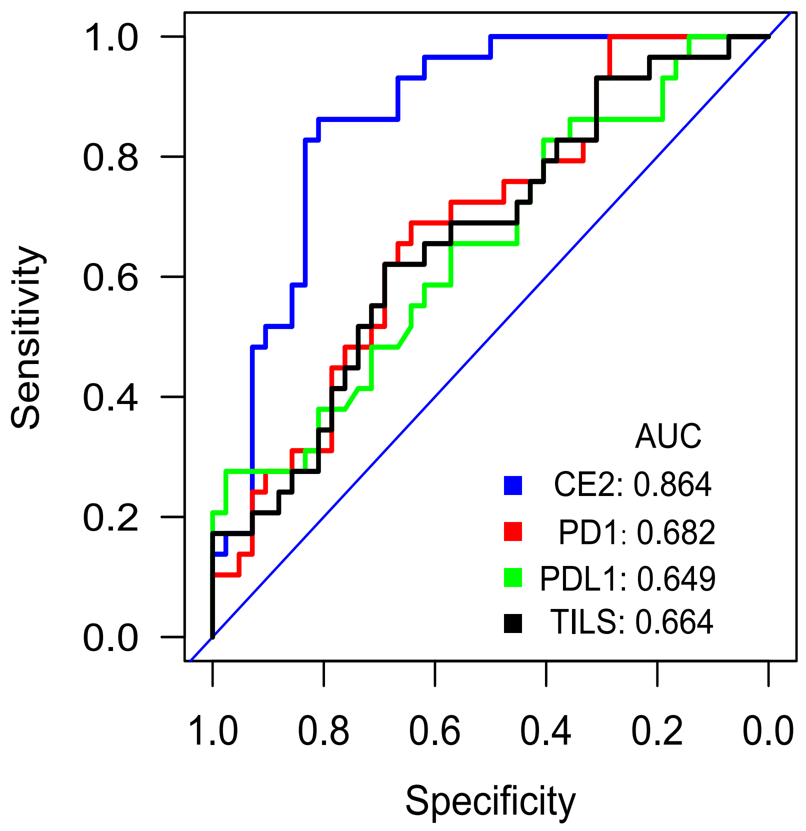

Sensitivity
Specificity
1.0
0.8
0.6
0.4
0.2
0.0
AUC
CE2: 0.864
PD1: 0.682
PDL1: 0.649
TILS: 0.664

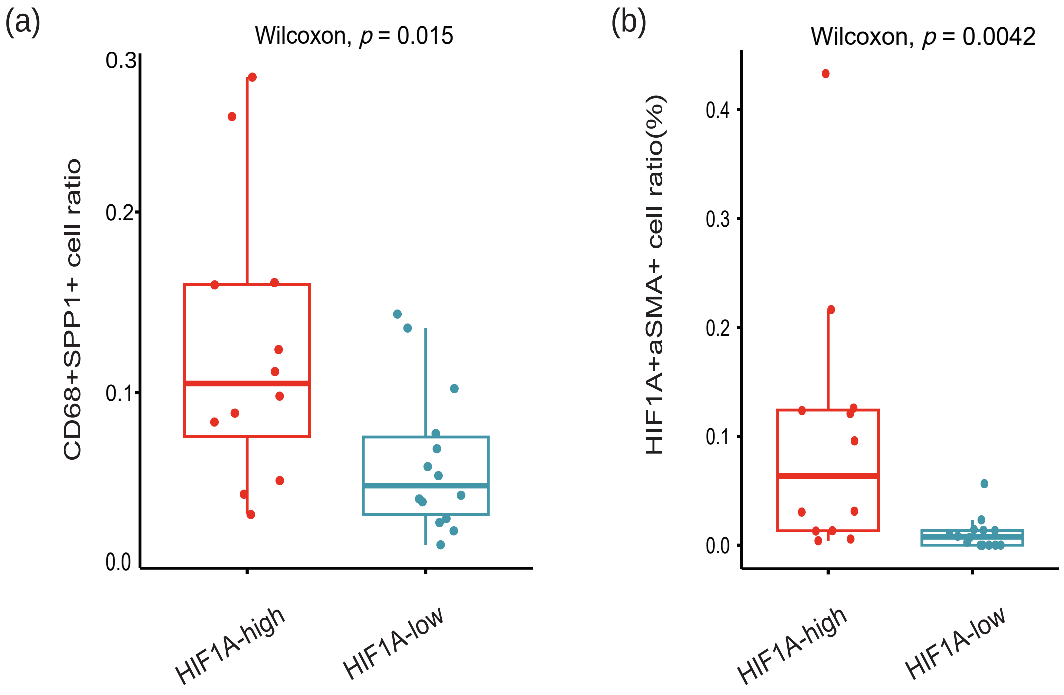

(a)
Wilcoxon, p = 0.015
CD68+SPP1+ cell ratio
0.3
0.2
0.1
0.0
HIF1A-high
HIF1A-low
(b)
Wilcoxon, p = 0.0042
HIF1A+aSMA+ cell ratio(%)
0.4
0.3
0.2
0.1
0.0
HIF1A-high
HIF1A-low
